# The uses of right heart catheterization in cardio-pulmonary disease: State-of-the-art

**DOI:** 10.1016/j.ahjo.2024.100488

**Published:** 2024-12-06

**Authors:** Bhavesh Katbamna, Lingling Wu, Mario Rodriguez, Phillip King, Joel Schilling, Jamal Mahar, Ajith P. Nair, Hani Jneid, Elizabeth S. Klings, Gerald L. Weinhouse, Sula Mazimba, Marc A. Simon, Markus Strauss, Chayakrit Krittanawong

**Affiliations:** a Division of Cardiovascular Disease, Section of Advanced Heart Failure and Transplant Cardiology, Barnes-Jewish Hospital, Washington University in St. Louis School of Medicine, USA; b Cardiovascular Division, the University of Alabama at Birmingham, Birmingham, AL, USA; c Section of Cardiology, Texas Heart Institute, Baylor College of Medicine, Houston, TX, USA; d John Sealey Centennial Chair in Cardiology, Chief of Cardiology, The University of Texas Medical Branch, TX, USA; e Pulmonary Center, Boston University School of Medicine, Boston, MA, USA; f Department of Medicine, Division of Pulmonary and Critical Care Medicine, Brigham and Women's Hospital, Boston, MA, USA; g Department of Cardiovascular Medicine, University of Virginia, Charlottesville, VA, USA; h Pulmonary Vascular Disease, a PHA Center of Comprehensive Care, Division of Cardiology, Department of Medicine, University of California San Francisco, San Francisco, CA, USA; i Department of Cardiology, Sector Preventive Medicine, Health Promotion, Faculty of Health, School of Medicine, University Witten/Herdecke, 58095 Hagen, Germany; j Department of Cardiology I- Coronary and Periphal Vascular Disease, Heart Failure Medicine, University Hospital Muenster, Cardiol, 48149 Muenster, Germany; k Cardiology Division, NYU Langone Health and NYU School of Medicine, New York, NY, USA

## Abstract

The right heart catheterization (RHC) remains an important diagnostic tool for a spectrum of cardiovascular disease processes including pulmonary hypertension (PH), shock, valvular heart disease, and unexplained dyspnea. While it gained widespread utilization after its introduction, the role of the RHC has evolved to provide valuable information for the management of advanced therapies in heart failure (HF) and cardiogenic shock (CS) to name a few. In this review, we provide a comprehensive overview on the indications, utilization, complications, interpretation, and calculations associated with RHC.

## Graphical abstract

**Figure:**
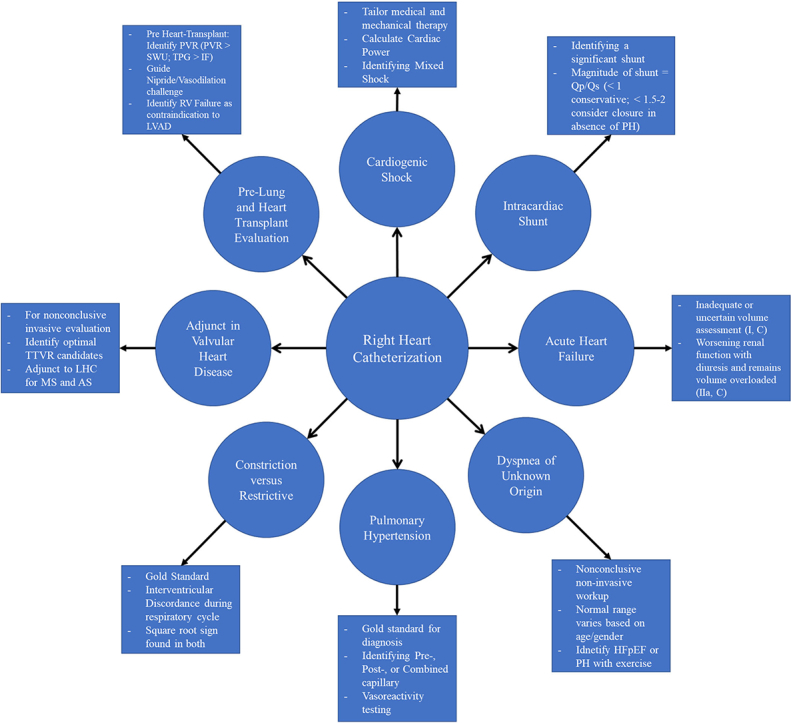

## 1 Introduction

The right heart catheterization (RHC) remains an important diagnostic tool, providing data on hemodynamics, cardiac output, and cardiopulmonary pressures to help clinicians make decisions in a variety of disease processes. With the development of the balloon floatation catheter (Swan-Ganz catheter) by Drs. Swan and Ganz in 1970, it attained widespread use without significant critical review due to its ability to obtain hemodynamic measurements at the bedside [1,2]. Several studies in the 1990's and early 2000's investigating the use of pulmonary artery catheters (PAC) suggested that it led to detrimental effects including increased length of stay, inconsistent interpretation of data, and increased mortality. Ultimately this resulted in a substantial decline in its use [3]. Despite such a dramatic change in utilization, it continues to play a key role in the diagnosis and management of pulmonary hypertension (PH), cardiogenic shock (CS), valvular heart disease, and evaluation prior to organ transplant, particularly of the heart and lungs. In this review, we discuss the indications, contraindications, complications, and interpretation of waveforms and measurements of RHC.

## 2 Epidemiology of utilization of RHC

The RHC was revolutionary with the availability of a balloon tipped, flow-directed catheter, giving clinicians the ability to place one at bedside and leave it in place for continuous monitoring. It achieved widespread use without empirical studies looking into the benefits or risks, and by the mid-1980s, up to 40 % of critically ill patients underwent RHC [4,5]. Concerns over this widespread utilization led to several studies which called the safety into question. Furthermore, several meta-analyses, observational studies, and randomized controlled trials have also shown no survival benefit from PAC use in critically ill patients. Ikuta et al. studied the national trends in PAC use and found a 67.8 % decline from 1999 to 2013. Specifically, there was a significant reduction in PAC placed for hospitalizations involving respiratory failure (92.3 % reduction) and acute myocardial infarction (74 % reduction). HF admissions on the other hand, demonstrated a 56 % reduction until 2009 [3].. Hernandez et al. demonstrated similar trends with a decrease in PAC use in HF from 2004 to 2007, followed by an increase in 2014. The authors also showed that use of PAC in CS has declined from 2004 to 2014. [6]

## 3 RHC indications

### 3.1 RHC in cardiogenic shock

With the ability to provide data on right and left sided filling pressures, CO, vascular resistance, and other hemodynamic parameters, the RHC provides valuable insight for the diagnosis and management of CS. Invasive hemodynamic monitoring can guide the use of vasoactive pharmacologic therapies and help determine when mechanical circulatory support (MCS) is necessary. Proponents state that invasive monitoring can guide therapy and optimize hemodynamics, however, several early studies showed a lack of benefit with use of the PAC in critically ill patients [7]. Several studies that investigated use of RHC or PAC in CS are summarized in Table 1 [7]. Cooper et al. set out to develop a noninvasive criterion for the diagnosis of CS and validated it against the PAC standard. Their noninvasive criteria utilized hypotension, hypoperfusion state, predisposition to CS, and echocardiographic findings and reported a sensitivity of 96.9 %, specificity of 90.8 %, positive predictive value of 64.6 %, and negative predictive value of 99.4 %. The authors suggested that these criteria be used to noninvasively exclude CS, while PAC be reserved to confirm CS in patients who meet criteria [8].

**Table 1:** Summary of studies investigating use of RHC in CS.

Study name and author	Primary indication	Outcomes	Comments
“SUPPORT”, Connors et al. [7]	Critically ill patients in the ICU: acute respiratory failure, multiorgan system failure, CHF	Patients with RHC vs no RHC: - Increased 30-day mortality (OR 1.24) - Increased LOS (14.8 days vs 13.0 days) - Increased resource utilization (49.3 vs 35.7 x $1000)	
“ESCAPE”, Binanay et al. [9]	Severe symptomatic heart failure despite recommended therapies	Use of PAC: - No difference in mortality (OR 1.26, 95 % CI 0.78 to 2.03) - Increased in-hospital adverse events (47 vs 25, *p* = 0.04) - No deaths related to PAC use	Excluded patients in cardiogenic shock requiring inotropes, vasopressors, or MCS
“PAC-Man”, Harvey et al. [10]	ICU patients; acute respiratory failure, multiorgan dysfunction, decompensated heart failure	Patients with PAC: - No difference in hospital mortality (68 % vs 66 %, *p* = 0.39) - Similar hospital and ICU LOS	No deaths related to PAC; Insertion related complications = 10 %
O'Neill et al. [11]	Acute myocardial infarction complicated by cardiogenic shock with Impella use	Use of PAC: - Improved survival rate of 63 % versus 49 % in patients with PAC use versus patients without PAC use	
“CardShock”, Sionis et al. [12]	CS	Use of PAC: - Mortality was 42 % compared to 34 % in non-PAC patients (*P* = 0.6)	Most common complication was VT during PAC insertion (5.6 %, 4/71)
“CSWG”, Garan et al. [13]	CS	Complete PAC hemodynamic data: - lowest in-hospital mortality compared to no PAC assessment (OR 1.57; 95 % CI 1.06 to 2.33) and to incomplete PAC data (OR 1.71; 95 % CI 1.29 to 2.25) - No significant difference between no PAC compared to incomplete PAC data	Complete PAC was defined as having all the following: PA systolic and diastolic pressure, PAWP, pulmonary artery saturation and right atrial pressure
Hernandez et al. [6]	CS and HF without CS	PAC use in CS - Lower mortality (35.1 % vs 39.2 %) PAC use in HF without CS: - Increased mortality (9.9 % vs 3.3 %)	National Inpatient Sample, Retrospective cohort study

In addition, PAC in patients with CS can help tailor management by identifying their unique hemodynamic profile e.g., a patient with a low cardiac index and high systemic vascular resistance (SVR) may benefit from afterload reduction strategies with agents such as Sodium Nitroprusside as opposed to utilizing an inotrope only strategy. This was further exemplified by Ranka et al. who showed that in patient's admitted with acute CS, RHC use was associated with a significant reduction in mortality in index admission by 31 %, a reduction in 30-day readmission rate, and a six-fold increase in left ventricular assist device (LVAD)/orthotopic heart transplantation during rehospitalization. The authors hypothesized that RHC allowed for early and accurate classification of CS and increased MCS or advanced therapy in cases of severe CS, resulting in improved outcomes [4,9]. Together these data suggest that RHC should be considered in CS that remains refractory to initial therapy or when advanced heart failure therapies are being considered.

In recent years, SCAI (Society of Cardiovascular Angiography&Interventions) definition and stages of cardiogenic shock and have been increasingly adopted in both research and clinical practices [10]. Through stage A (at risk) to stage E (extremis), this new definition highlights the dynamic nature of the CS as well as the importance of timely recognition and interventions to improve the outcome of patients with CS. Right heart catheterization is helpful in both evaluation and prognostication of CS as described above. When there is concern for full blown CS, invasive hemodynamic measuring with cardiac index <2.2 L/min/m2 and pulmonary artery wedge pressure (PAWP) >15 mmHg remains to be gold standard for diagnosis. In addition, Cardiac power output (CPO) and Pulmonary artery pulsatility index (PAPI) are also useful hemodynamic index to identify the severe LV dysfunction and RV failure, respectively [11,12].

Right heart catheterization and invasive hemodynamic monitoring have been recommended for guidance of utilization of MCS when treating CS, as clinical assessment of PAWP and CVP are often inaccurate and unreliable [13]. All cardiogenic shock has decreased cardiac output, but they may have different hemodynamic profiles. In the setting of predominantly elevated left side filling pressure, an unloading device such as percutaneous LVAD can help decrease PAWP and improve CVP by reducing RV afterload. However, when there is commitment right sided congestion that is not responding to the LV unloading, diuresis or CRRT might be indicated to further improve the cardiac output. When there is more predominant RV failure evidenced by marked elevated CVP and RV dysfunction, pRVAD might be indicated for RV unloading. In the face of severe and rapid cardiopulmonary collapse, VA-ECMO is often used to restore organ perfusion. However, its use among CS patients with severe LV dysfunction may further increase left side filling pressure and cause pulmonary edema. pLVAD such as Impella can be added to the VA-ECMO to further unload the LV and improve the hemodynamics [14]. Invasive hemodynamic monitoring has been shown to be associated with improved outcome of CS patients who received MCS. In a large retrospective study of 1414 patients with CS, the use of complete PAC-derived hemodynamic data prior to MCS initiation is associated with improved survival from CS [15].

### 3.2 RHC in pulmonary hypertension

RHC remains the gold standard to diagnose, risk stratify and classify PH. The European Society of Cardiology (ESC) and European Respiratory Society (ESR) guidelines recommend use of RHC to investigate when echocardiography findings demonstrate high probability of PH to determine the etiology and confirm the diagnosis, and to support treatment decisions [16]. Additionally, RHC is recommended in patients with intermediate probability of PH by echocardiography and unexplained signs or symptoms of PH, particularly if they have co-morbidities that increase their risk of PH. Echocardiogram has been shown to have good correlation but only moderate precision of estimating the Pulmonary artery systolic pressure (PASP) value calculated from TR velocity [17]. The errors in measurement of peak TRV signal can lead to under or overestimate of PASP. In a large study of 1695 patients who had both echocardiogram and RHC, echocardiogram was found to have sensitivity of 87 % and specificity of 79 % for PASP cut off value of 36 mmHg [18]. Due to this limitation, echocardiographic information can only grade the probability of PH being present rather than making the diagnosis [19].

The World Health Organization (WHO) classification categorizes PH into 5 groups based on the specific pathophysiology and response to treatment: Group 1: pulmonary arterial hypertension [PAH], Group 2: PH due to left heart disease; Group 3: PH due to lung disease and/or hypoxia; Group 4: PH due to pulmonary artery obstructions; Group 5: PH with unclear and/or multi-factorial mechanisms [20]. Table 2 provides an overview of the classifications. Prior to 2019, PH was defined as a mean pulmonary artery pressure (mPAP) ≥ 25 mmHg at rest which was arbitrarily defined. A retrospective analysis of all normative published RHC data by Kovacs et al. found that supine mPAP at rest in healthy individuals was 14.0 ± 3.3 mmHg and two standard deviations above the mean (20.6 mmHg), would represent the upper limit of normal [21]. According to ESC 2022 Guidelines, PH is defined as a mPAP > 20 mmHg. The Task Force included a pulmonary vascular resistance (PVR) ≥ 2 Wood units (WU) and a PAWP ≤ 15 mmHg to define all forms of pre-capillary PH [20]. The hemodynamic definitions can help with determining the diagnostic groups as outlined in Table 3. Currently, there are no treatments approved for mPAP between 20-24 mmHg and PVR between 2-3 WU.

**Table 2:** Overview of the groups of pulmonary hypertension [98].

Classification of Pulmonary Hypertension
Group 1: Pulmonary Arterial Hypertension (PAH) 1.1 Idiopathic 1.2 Heritable PAH 1.3 Drug- and toxin-induced PAH 1.4 PAH associated with 1.4.1 connective tissue disease 1.4.2 HIV infection 1.4.3 portal hypertension 1.4.4 congenital heart disease 1.4.5 schistosomiasis 1.5 PAH long-term responders to CCB 1.6 PAH with overt features of venous/capillaries (PVOD/PCH) involvement 1.7 Persistent PH of the newborn syndrome
Group 2: PH due to left heart disease 2.1 Heart failure with preserved LVEF 2.2 Heart failure with reduced LVEF 2.3 Valvular heart disease 2.4 Congenital/acquired cardiovascular conditions leading to post-capillary PH
Group 3: PH due to lung diseases and/or hypoxia 2.1 Obstructive lung disease 2.2 Restrictive lung disease 2.3 Other lung disease with mixed restrictive/obstructive pattern
Group 4: PH due to pulmonary artery obstructions 2.1 Chronic thromboembolic PH 2.2 Other pulmonary artery obstructions
Group 5: PH with unclear and/or multifactorial mechanisms 2.1 Hematological disorders 2.2 Systemic and metabolic disorders 2.3 Others 2.4 Complex congenital heart disease

**Table 3:** Hemodynamic findings to categorize PH [98].

Type of PH	mPAP	PAWP	PVR
Pre-capillary (groups 1, 3, 4 and 5)	>20 mmHg	≤15 mmHg	≥2 WU
Isolated post-capillary (groups 2 and 5)	>20 mmHg	>15 mmHg	<2 WU
Combined pre- and post-capillary (groups 2 and 5)	>20 mmHg	>15 mmHg	≥2 WU

Key hemodynamic measurements obtained from RHC that categorize PH.

PH = pulmonary hypertension, mPAP = mean pulmonary artery pressure, PAWP = pulmonary artery wedge pressure, PVR = pulmonary vascular resistance, WU = Wood units.

In patients with newly diagnosed PAH, vasoreactivity testing is recommended in those with idiopathic, heritable or drug/toxin induced PAH only to assess for possible treatment response with calcium channel blockers [22]. Inhaled nitric oxide (10–20 ppm) or intravenous epoprostenol are the two preferred agents which can be utilized in this setting. Based on data by Sitbon et al. a positive vasodilator response was defined as a reduction in mPAP ≥10 mmHg to reach a mPAP ≤ 40 mmHg with an increased or unchanged CO [22]. Only 10 % of idiopathic PAH patients will be vasoreactive.

RHC is an important part of mortality risk stratification in PAH [22]. Elevated right atrial pressure and reduced cardiac index have repeatedly been demonstrated to predict increased mortality risk in PAH. Elevated right atrial pressure is included in the REVEAL 2.0 risk score (Benza et al. CHEST 2019), a highly validated risk score that can help guide therapy. In patients with PAH, repeat right heart catheterizations are often utilized to determine the response to therapy.

Even with hemodynamic measurements, it can be challenging to distinguish pre- and post-capillary PH in the setting of HF with preserved ejection fraction (HFpEF) or optimal volume status. Evocotive manuevers, such as exercise and fluid challenge, can be very useful to distinguish HFpEF for PH as a cause of dyspnea. The ratio of change in PAWP per change in CO >2 mmHg/L/min can be used to identify HFpEF (Greg Lewis et al. and also see 2022 PH guidelines). The saline challenge protocol can help differentiate Group 1 PAH from Group 2 PH and involves intravenous administration of a 500 mL 0.09 % saline bolus over 5 to 10 min [21]. Although normal subjects can have an increase in filling pressures with a saline bolus, those with HFpEF had a steeper rise in PAWP comparatively [23]. Several studies have demonstrated that a PAWP >18 mmHg after saline challenge is suggestive of post-capillary PH [[23], [24], [25], [26]]. D'Alto et al. utilized the saline challenge to correctly reclassify five out of 66 patients without PH and eight out of 124 with pre-capillary PH into post-capillary PH [25]. Left ventricular end-diastolic pressure (LVEDP) and PAWP are often used interchangeably to estimate left-sided filling pressures. However, several studies have demonstrated discrepancies between these two measurements leading to misclassification of PH group. PAWP underestimates LVEDP in older age and overestimates LVEDP in atrial fibrillation, rheumatic valve disease, or those with a larger left atrial diameter. If RHC and PAWP were used to classify PH group in the above situations, it would misclassify nearly 30 % of patients [27]. Therefore, ESC/ESR guidelines recommend use of LVEDP when PAWP is unreliable [16]. Some authors have advocated for earlier use of RHC in the diagnostic schema of PH than some societal guidelines have recommended [19].

### 3.3 RHC in unexplained dyspnea

Invasive cardiopulmonary exercise testing (iCPET) can be a valuable tool in evaluating unexplained dyspnea, especially when non-invasive workup is inconclusive. iCPET allows for the measurement of physiological data that facilitates diagnosis of exercise-induced PAH, exertional HFpEF, or preload dependent limitations to CO [28,29]. It is also performed to measure the contribution of cardiac and lung pathology to a patient's symptoms in multifactorial disease and to determine the severity of cardiac limitations in heart failure with reduced ejection fraction [30]. When performing iCPET, RHC and sometimes radial artery catheterization (vrs pulse oximetry) are obtained to measure cardiac hemodynamics and peripheral tissue oxygen consumption. Caution needs to be used in patients with significant arterial insufficiency or Raynaud's phenomenon as radial artery catheterization can induce acute limb ischemia. An upright or semi-supine cycle ergometer, treadmill, or arm ergometer are utilized until the resistance is increased and the patient is exhausted. The pulmonary arterial pressure, right atrial pressure, and mean arterial pressure are measured continuously, while PAWP, arterial blood gases, and lactate are measured every minute. Baseline ventilation and pulmonary gas exchange measurements are obtained and repeated throughout exercise testing [28,29].

Herve et al. investigated the accuracy of mPAP alone compared to mPAP combined with peak exercise total PVR (TPVR) and supine dynamic exercise testing and found that the combination of maximal mPAP >30 mmHg and TPVR >3 WU corresponded to a similar sensitivity (0.93) but an improved specificity from 0.77 to 1.0 when distinguishing healthy subjects from ones with exercise-induced PH. The authors, however, were unable to accurately differentiate if PH was caused by left heart disease or pulmonary arterial disease [31]. While exercise PH has been reintroduced in the 2022 PH guidelines, there remain no proven therapies. In patients with PAH and HFpEF, Santos et al. hypothesized that exercise upright RHC would provide additional information for the diagnosis of HFpEF and PAH in patients with unexplained exertional dyspnea. Patients had same day supine resting RHC followed by iCPET. 212 patients had normal supine resting RHC, however, 46 (22 %) of these patients had abnormal iCPET results. The use of iCPET reclassified diagnosis for 16 (43 %) of patients. 12 (40 %) patients with HFpEF had normal cardiac profile and 4 (31 %) of patients with PAH had no intrinsic pulmonary vascular disease found with iCPET [32]. This study highlighted the hemodynamic changes in relationship to position during iCPET which may alter the interpretation of the result [33].

Although diagnostic criteria for exercise-induced PAH have been attempted, they remain controversial as normal ranges for mPAP are highly variable and dependent on age [28]. Kovacs et al. found that there were no significant association with age and resting mPAP, however, mPAP during exercise was independently linked to age with nearly half of the subjects ages ≥50 years exceeding an exercise mPAP >30 mmHg. The authors also found that nearly 20 % of patients younger than 50 years of age had mPAP >30 with maximal exertion, making it difficult to define normal ranges during exercise [21]. Cardiopulmonary exercise testing has proved to be useful in differentiating cardiopulmonary causes of dyspnea from others. Elevated PAWP/CO slope during exercise (>2 mmHg/L/min) is common in patients with dyspnea on exertion with normal wedge pressure at rest and predicts exercise capacity and heart failure outcomes [28].Single measurements at rest might be insufficient and exercise measurements may refine the HFpEF diagnosis.

Due to the complex procedures and requirement of muti-specialty collaboration. The iCPETs are usually performed at larger referral centers. In a retrospective study of iCPET center at Brigham and Women's hospital, 530 of 864 referrals were investigated with iCPET, over a span of 3 years [34]. Exercise PAH, HFpEF, dysautonomia, oxidative myopathy accounted for 16.6 %, 17.7 %, 21.1 % and 24.5 % of the final diagnoses, respectively [34].

### 3.4 RHC in constrictive pericarditis and restrictive cardiomyopathy

Constrictive pericarditis is associated with equalization of diastolic pressures in all chambers, impaired ventricular filling, and signs or symptoms of right heart disease. As restrictive cardiomyopathy can present with similar findings, it is important to differentiate these entities to determine whether pericardial stripping may be indicated. The diagnosis can be challenging, and differentiation between the two requires a comprehensive evaluation with physical examination, computed tomography, magnetic resonance imaging, echocardiographic, and hemodynamic findings. In constrictive disease, rapid ventricular filling occurs early in the cycle followed by a rapid increase in pressure due to the noncompliant pericardium. This results in a rapid “y” descent on the atrial pressure waveform and a “square root” sign on ventricular pressure waveform, which may also be noted on pressure tracings of restrictive cardiomyopathy [35].

The most reliable hemodynamic signs of constrictive pericarditis are those related to respiratory variation in RV versus LV filling. Constrictive physiology is demonstrated by significant ventricular discordance during the respiratory cycle, whereas concordance of ventricular pressures is the rule in restrictive cardiomyopathy [36]. There have been several criteria to help differentiate constrictive and restrictive disease, but they have poor sensitivity and specificity. The systolic area index is a ratio of the RV area to LV area in inspiration versus expiration, and a value >1.1 is 97 % sensitive and 100 % specific for identification of constrictive pericarditis [35,37,38]. Other findings that differentiate the two can be found in Table 4.

**Table 4:** Comparative Findings of Constrictive Pericarditis and Restrictive Cardiomyopathy [35,37,38].

	Constrictive Pericarditis	Restrictive Cardiomyopathy
Traditional findings		
End-diastolic pressure equalization	LVEDP-RVEDP ≤5	LVEDP-RVEDP ≥5
Dip and plateau morphology	Prominent rapid diastolic filling waves on LV tracings (≥5 mmHg; square-root sign)	LV rapid filling wave <5 mmHg
Right Ventricular Systolic Pressure	< 55 mmHg	> 55 mmHg
Right ventricular end-diastolic pressure (RVEDP)	RVEDP ≥1/3 RV systolic pressure	RVEDP <1/3 RV systolic pressure
Kussmaul's sign	Failure of RA pressure to drop upon inspiration.	Normal respiratory variation in mean RAP

Dynamic findings		
Dissociation of intrathoracic-intracardiac pressures	A difference ≥ 5 mmHg between expiratory and inspiratory gradients between the PAWP and LV early diastolic pressures	Concordant variation with respiration yielding to a difference between gradients of <5 mmHg
Ventricular interdependence	An inspiratory/expiratory systolic area index ≥1.1	An inspiratory/expiratory systolic area index<1.1

### 3.5 RHC in pre heart and lung transplant workup

RHC is an essential part of the evaluation for heart transplant. The International Society for Heart and Lung Transplantation (ISHLT) Guidelines recommend that RHC should be performed in all adult candidates prior to listing for cardiac transplant (Class 1, level of evidence C). Once patients are listed, RHC should be repeated at 3-to-6-month intervals, most notably in the setting of reversible PH or worsening HF symptoms [39,40].

The presence of elevated PVR or PH that is deemed irreversible with vasodilators remains a contraindication to isolated heart transplant, as the donor right ventricle may fail in the operating room or immediately postoperatively in the face of high PA pressures [41]. Candidates with PVR > 5 WU and transpulmonary pressure gradient (TPG) > 15 mmHg have an increased 30-day mortality, and RHC remains crucial to recognizing these individuals [42]. In patients with systolic PAP ≥ 50 mmHg and either PVR > 3 WU or TPG ≥ 15 mmHg, a vasodilator challenge with nitroprusside, IV prostanoids, or inhaled nitric oxide is indicated. Patients deemed to be reasonable candidates for transplantation are able to achieve a PVR of ≤3 WU while keeping systolic blood pressures above 85 mmHg [39]. If the vasodilator challenge fails and the patients significantly decompensate, as evidenced by elevated PAWP and central venous pressure (CVP), they should be hospitalized for continuous hemodynamic monitoring and optimized with diuretics, inotropes, and vasoactive agents for 24 to 48 h. If medical therapy fails to reverse PH, consideration can be given for a trial of termporary MCS or intra-aortic balloon pump although having a clear endpoint for support is needed. Durable LVAD may be considered in patients otherwise felt to be candidates (including careful attention to RV function) and in some cases longterm LV unloaded may result in improved PVR [double check literature on this]. It is also reasonable to trial milrinone for 3 to 6 months and then reevaluate hemodynamics. Hemodynamics should be measured at 3 to 6 months after LVAD according to ISHLT guidelines (Class IIA, Level of Evidence: C). When both medical therapy and MCS fail, these patients are categorized as irreversible PH and isolated heart transplant remains contraindicated [39]. Klotz et al. attempted to reduce PVR < 2.5 WU and TPG < 13 mmHg in patients being evaluated for heart transplant using prostaglandin E1 (PGE-1). The 1-year mortality after heart transplant was 22 % in the PH group treated with PGE-1, while it was 14 % in patients without any PH. There was no statistical difference in survival between these groups [43]. One of the indications for combined heart-lung transplant includes secondary pulmonary hypertension (in many cases related to congenital heart disease) that does not respond to therapy, inotropes, and/or MCS, however combined heart-lung transplant is only performed in a limited number of centers and organ scarcity has prevented any considerable volumes of this procedure. The RHC also plays an important role prior to listing for lung transplantation. The Lung Allocation Score is utilized to waitlist patients based on mortality and posttransplant survival [43].. While the LAS takes some hemodynamic metrics of PH into consideration, PAH patients remain at a disadvantage in getting organs.

### 3.6 RHC in other solid organ transplant evaluation

Right heart catheterization has been also utilized in solid organ transplantation other than heart and lung for pretransplant evaluation. For orthotopic liver transplantation (OLT), guideline recommends mandatory screening for portopumonary hypertension (PoPH) with echocardiogram in all OLT candidates [44]. For patients with elevated PASP (≥40 mmHg), RV dysfunction, LV dysfunction or significant valvular disease, RHC is indicated for evaluation degree and the type of pulmonary hypertension to further assess the candidacy of liver transplant [45]. Brachial vein might be a preferred access site for RHC for patients with severe coagulopathy [45]. For renal transplantation, the echocardiogram is indicated for patients who have been on dialysis for >2 years, has risk factors for pulmonary hypertension [46]. For those who have an estimated pulmonary systolic pressure >45 mmHg should be referred for further evaluation with right heart catheterization [46]. All patients being considered for pancreas should be evaluated with echocardiogram. For candidates with right ventricular systolic pressure (RVSP) >50 mmHg, a right heart catheterization is also recommended [47].

### 3.7 RHC in detection and quantification of shunts

An assessment of oxygen saturation of the superior vena cava (SVC) and the main pulmonary artery (PA) should be performed during RHC to detect presence of a shunt. A significant step-up in blood oxygen is defined as an increase in blood oxygen content or saturation that exceeds the normal variations of the right heart in the absence of a shunt [[48], [49], [50]], and a step-up in oxygen saturation of >8 % should prompt further investigation with stepwise assessments of oxygen saturation, also known as detailed sampling [51,52]. Upon confirming the presence of a significant step-up, measurements are obtained with fluoroscopic guidance and pressure measurement from the SVC (low and high), inferior vena cava (IVC) [low and high], right atrium (RA) [high, mid, and low], right ventricle (RV) [outflow tract, mid, apex], right ventricular outflow tract, main PA, left and right PA, and left ventricle (LV) as well as distal aorta in selected patients [52]. The criteria for a significant step-up in oxygen saturation or oxygen content to signify a shunt are displayed in Table 5 [[52], [53], [54]]. The magnitude of the shunt is then provided by the ratio of pulmonic flow to systemic flow (Qp/Qs), such that a Qp/Qs < 1 is managed conservatively, while a Qp/Qs > 1.5 warrants surgical or percutaneous repair [52,55,56].

**Table 5:** Oxygen saturation associated with significant shunts [[52], [53], [54]].

Criteria for Step-Up	Level of Shunt		
	Atrial (SVC or IVC to RA)	Ventricular (RA to RV)	Great Vessel (RV to PA)
Δ Mean O2 %Sat (distal and proximal chamber samples)	≥7 %	≥5 %	≥5 %
Δ Highest O2 %Sat (distal and proximal chamber samples)	≥10 %	≥5 %	≥8 %

### 3.8 RHC in valvular heart disease studies

The evaluation of valvular heart disease using invasive hemodynamics has fallen out of favor largely due to improvements in non-invasive imaging. When echocardiography and other noninvasive imaging is inconclusive, RHC can be considered per the ESC guidelines [57]. Below are the following instances where RHC may prove to be useful in valvular disease:

#### 3.8.1 Aortic stenosis

In aortic stenosis, coronary angiography in addition to RHC is often utilized to evaluate for existing coronary artery disease and PH as part of the pre-transcather aortic valve replacement evaluation [58].

#### 3.8.2 Mitral stenosis

RHC is indicated in patients with mitral stenosis who have symptoms of PH or when non-invasive measurements of the mitral valve are inconsistent. Simultaneous measurement of the PAWP and LV pressure are used to evaluate the trans-mitral gradient [59]. The PAWP will frequently overestimate the trans-mitral gradient by 30 % to 50 %, as it does not account for a delay of transmission of pressure through the pulmonary circulation [60].

#### 3.8.3 Tricuspid regurgitation

Stocker et al. showed that evaluation by invasive hemodynamic monitoring plays an important role in identifying patients with severe tricuspid regurgitation who would benefit from transcatheter tricuspid valve repair (TTVR). Patients were stratified into 3 groups by hemodynamic measurements to examine post-operative outcomes and mortality after TTVR. Expectedly, patients without any PH (mPAP <30 mmHg) and post-capillary PH (mPAP >30 mmHg, TPG ≤17 mmHg) demonstrated better post-operative outcomes and 1-year survival rate compared to patients with pre-capillary PH (mPAP >30 mmHg, TPG >17 mmHg). PAP, TPG, PVR, RV stroke work were predictors of all-cause mortality after valve repair, while PAWP, RAP, CO, and pulmonary artery pulsatility index had no significant association. The authors suggest invasive hemodynamic assessments should be used to stratify patients to select for TTVR [61].

### 3.9 RHC in the diagnosis of lymphangitic carcinomatosis

The use of blood drawn from a PAC in the wedge position may be useful in evaluating for lymphangitic carcinomatosis in patients who are unable to undergo invasive procedures, such as a lung biopsy. Masson et al. collected blood drawn through a wedged PAC and examined the cytologic characteristics in eight patients with lymphangitic carcinomatosis and 40 control patients divided into two groups (23 without cancer but various pulmonary disorders and 17 with cancer but without pulmonary metastases). Seven out of eight patients with lymphangitic carcinomatosis were found to have malignant cells on pulmonary microvascular cytology. The authors suggested this as an alternative method when a RHC is being performed for hemodynamic assessment or lung biopsy is too risky [43].

## 4 RHC contraindications

Absolute Contraindications: presence of right-ventricular assist device, tricuspid or pulmonic valve endocarditis, and skin insertion site infection [[62], [63], [64]].

Relative Contraindications: severe acidemia (pH <7.1), and bioprosthetic tricuspid or pulmonic valve [[62], [63], [64]].

Mechanical tricuspid valve has been traditionally thought to be absolute contraindication for RHC due to the risk of catheter entrapment, prosthetic vale damage or thrombosis formation, but it has been increasingly reported it can be safely performed in selected cases with precautions [65].

Presence of left bundle branch block: a transient right bundle branch block induced by the catheter can lead to complete heart block. Although rare, placement of a standby pacemaker catheter is recommended [61].

Coagulopathy: There are limited recommendations in the setting of coagulopathy which can also be corrected with transfusion. In a study by Betz et al., the bleeding complications were compared between patients with an INR < 1.8 and INR ≥1.8 (range 1.8–4.0). The overall bleeding complications were extremely rare occurring in 0.6 % (2/312) of patients, both who developed a hematoma requiring prolonged manual compression and belonged in the INR < 1.8 group [61].

## 5 RHC complications

RHC is an invasive procedure, and as with any procedure there are complications associated with each step. In the ESCAPE trial, only a total of 9 (4.2 %) procedural complications occurred, and none were associated with in-hospital deaths. These included infection (4 patients), bleeding (2 patients), catheter knotting (2 patients), pulmonary infarction (1 patient), pulmonary hemorrhage (1 patient), and ventricular tachycardia (1 patient) [66]. PAC-Man (Pulmonary Artery Catheters in Patient Management in Intensive Care) demonstrated a complication rate of 9.5 % (46 patients) which included hematoma (4 %), arterial puncture (3 %), and arrhythmias (3 %). Similar to the ESCAPE trial, these complications were not fatal [67]. A combined 5-year retrospective and 6-month prospective analysis looked at the number of adverse events and complications associated with RHC. Out of a total of 7218 RHC performed, there were 76 serious adverse (1.1 %) events during the study period. Seventy-two of these serious adverse events were non-fatal and twenty-one (28 %) of these non-fatal events resulted in a hospital admission or prolonged stay. The most common complications were related to venous access followed by arrhythmias. There were only four (0.055 %) procedure-related deaths during the study period [68]. With the widespread use of point of care ultrasound in nearly all critical care settings, the rate of complication associated with insertion has improved compared to when PAC-Man and the latter study were published-2005 and 2006 respectively.

Complications with venous access include hematoma at insertion site, arterial puncture, pseudoaneurysm formation, pneumothorax, thoracic duct injury, or thrombosis. The rate of thrombosis varies on the insertion site, with subclavian access having the lowest thrombotic risk at 1.9 % [69] and length of time left indwelling. The rate of pneumothorax is lower with the use of the internal jugular veins for insertion compared to the subclavian veins and obviously, non-existent with a femoral vein insertion [69]. The next set of complications are related to insertion of the PAC and include arrhythmias, catheter knotting, and PA rupture. Cardiac arrhythmias from PAC are mostly transient and include premature atrial or ventricular contractions, although sustained ventricular and superventricular arrhythmias may occur. Right bundle branch block can occur in up to 5 % of PAC insertions, therefore, patients with pre-existing left bundle branch block (LBBB) are at a risk of a complete heart block making LBBB a contraindication for RHC unless immediate pacing is available during the procedure [7,[70], [71], [72]].

The most feared complication of RHC is PA rupture, and although the incidence remains low at 0.03 % to 0.2 %, it has a mortality rate of 70 %. When a patient with a PAC develops new onset hemoptysis, rupture of the PA should be suspected. It can be complicated by hemothorax or endobronchial hemorrhage and require emergent surgical approach such as a thoracotomy [71,73]. PA rupture occurs from over-inflation of the PAC balloon or migration of the catheter tip [74], and risk factors include severe pulmonary hypertension, age > 60, use of anticoagulation, cardiopulmonary bypass, and mitral valve disease [7,73,75]. In a study by Kearney and Shabot, PA rupture was seen in ten patients out of 32,442 (0.03 %) who required hemodynamic monitoring with PAC. All ten patients had hemoptysis and seven died. The authors found that emergent thoracotomy was essential for survival in the presence of hemothorax [73]. In order to minimize risk, a chest x-ray should always be obtained before and after adjustment of the PAC, and fluoroscopy may be preferrable for PAC insertion in certain situations. Weinberg et al. showed that insertion under video fluoroscopy allows for positioning of the catheter with less time, fewer attempts, and decreased rate of complications compared to PAC floated by real-time pressure waveform monitoring [76]. It is also important to adopt safe techniques when using PAC. Avoid inflating the ballon in the distal pulmonary artery or against pressure. In case of suspected PA vascular injury due to right heart catheterization that resulted in significant bleeding, it is important to first secure the airway, often with endotracheal intubation. Acute hemostasis can be achieved by inserting a new Swan-ganz catheter into the same PA branch and inflating the ballon proximally to the bleeding site [77]. The PA rupture can be treated endovascularly with stents, coil embolization or vascular plug or surgically with lobectomy [78,79]. Indwelling PAC increased the risk of catheter site infection, bacteremia/sepsis, or right-sided endocarditis. Rowley et al. studied the frequency of right-sided endocardial lesions in catheterized patients. One or more right-sided endocardial lesions were found in 29 of the 55 catheterized patients, with four (7 %) of these lesions representing infective endocarditis while the remaining were uninfected. The authors observed infective and non-infective vegetations were in similar locations, including the pulmonic valve and right atrium, which would suggest it was a consequence of direct endocardial damage from the PAC. The length of time the catheter was in place was not significantly related to the frequency of the lesions occurring [80]. Al-Hijji et al. studied the major adverse events from left heart catheterization (LHC) and LHC combined with RHC using the electronic medical record and ICD-9 codes. The combined risk of death, myocardial infarction, stroke, coronary dissection, pericardial effusion, or cardiac tamponade was 8.2 out of 10,000 procedures. Furthermore, adding a RHC to a LHC procedure did not increases risk of these complications [81]. Pulmonary infarction was described by Foote et al. to be a result of thrombi formed around the catheter or catheter occlusion of the small pulmonary artery. Pulmonary infarction was found in 9 % of patients, and they recommended frequent chest x-ray to monitor for distal migration of the catheter tip [81]. In another study, pulmonary infarction was found in 2 out of 112 RHC and both resulted from distal migration and pulmonary artery occlusion by the catheter [81]. This necessitates monitoring with daily chest x-ray to lessen the risk. Overall, the rate of complications of PAC and RHC is low in the hands of an experienced provider.

## 6 What do guidelines say?

### 6.1 The American College of Cardiology/American Heart Association HF guidelines [82]

Class I:

Invasive hemodynamic monitoring with a pulmonary artery catheter should be performed to guide therapy in patients who have respiratory distress or clinical evidence of impaired perfusion in whom the adequacy or excess of intracardiac filling pressures cannot be determined from clinical assessment. (Level of Evidence: C)

Class IIa:

Invasive hemodynamic monitoring can be useful for carefully selected patients with acute HF who have persistent symptoms despite empiric adjustment of standard therapies and:

1. whose volume status, perfusion, or systemic or pulmonary vascular resistance is uncertain;
2. whose systolic pressure remains low, or is associated with symptoms, despite initial therapy;
3. whose renal function is worsening with therapy;
4. who require parenteral vasoactive agents; or
5. who may need consideration for MCS or transplantation. (Level of Evidence: C)

Class III: No Benefit

Routine use of invasive hemodynamic monitoring is not recommended in normotensive patients with acute decompensated HF and congestion with symptomatic response to diuretics and vasodilators. (Level of Evidence: B).

### 6.2 European Society of Cardiology (ESC) and the European Respiratory Society (ERS) [16]

Class I:

1. Patients with unexplained exertional dyspnea, syncope, and/or signs of right ventricular dysfunction should be screened with transthoracic echocardiography followed by confirmed diagnosis with RHC
2. RHC is required when treatment for PH is being considered. RHC is required to confirm diagnosis of pulmonary hypertension. It is required for diagnosis of PAH and Chronic thromboembolic pulmonary hypertension.
3. RHC is recommended in all cases of suspected PAH due to connective tissue disease
4. RHC is recommended for patients with congenital cardiac shunt to consider surgical correction
5. RHC is recommended in patients with PH due to left-sided heart disease (Group 2 PH) or chronic lung disease (Group 3 PH) if being considered for organ transplantation
6. High doses of CCBs are recommended in patients with idiopathic PAH, heritable PAH, and drug-induced PAH who respond to acute vasoreactivity testing. Reassessment after 3–4 months of therapy with RHC is recommended after treatment [16]

Class IIa:

1. RHC should be considered in pulmonary arterial hypertension (Group 1 PH) to assess the treatment effect of medications

### 6.3 The International Society for Heart and Lung Transplantation Guidelines [16]

*Class I, Level of Evidence C:*

1. RHC should be performed on all candidates in preparation for listing for cardiac transplantation and annually until transplantation
2. RHC should be performed at 3- to 6-month intervals in listed patients, especially in the presence of reversible pulmonary hypertension or worsening of heart failure symptoms)
3. A vasodilator challenge should be administered when the pulmonary artery systolic pressure is ≥50 mmHg and either the transpulmonary gradient (TPG) is ≥15 or the pulmonary vascular resistance (PVR) is >3 Wood units while maintaining a systolic arterial blood pressure > 85 mmHg
4. When an acute vasodilator challenge is unsuccessful, hospitalization with continuous hemodynamic monitoring should be performed, as often the PVR will decline after 24 to 48 h of treatment consisting of diuretics, inotropes and vasoactive agents such as inhaled nitric oxide.

## 7 RHC vascular accesses

Venous access may be dependent on a variety of factors including operator experience, presence other cardiac devices, patient anatomy, and prior complications. Traditionally, femoral vein was used if a left heart catheterization was performed at the same time as a RHC.

Internal jugular vein access is commonly used for patients in the critical care setting for continuous hemodynamic monitoring, and it is easier to manipulate into the PA compared to femoral vein access. Ultrasound is utilized for cannulation and a double Seldinger technique allows for lower risk of bleeding complications [7]. Brachial vein access can also be utilized. This can easily be done by swapping out a 20G venous catheter and over the wire exchanging it for a 5–8 F sheath.

## 8 RHC measujrements and interpretation of the results

### 8.1 Measurements and waveforms

**Fig. 1:**
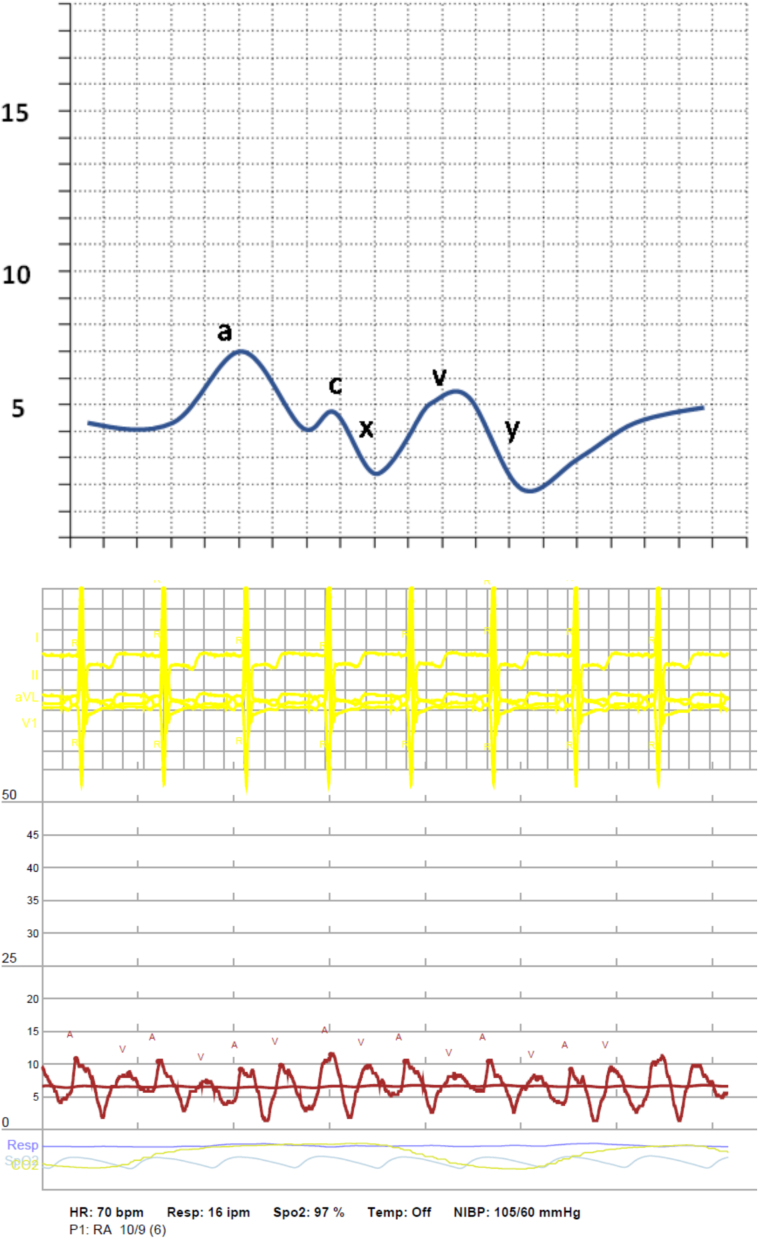
Normal Waveform RA Pressure (Normal RA pressure waveform is described by the “a”, “c”, and “v” waves and “x” and “y” descents. The a wave represents atrial systolic. The c wave occurs with closure of the tricuspid valve. The x descent shows atrial relaxation with atrial diastole. The v wave is associated with rise in RA pressure due to RA filling during ventricular systole. Finally, the y descent occurs during RA emptying in early diastole when rapid ventricular filling occurs).

**Fig. 2:**
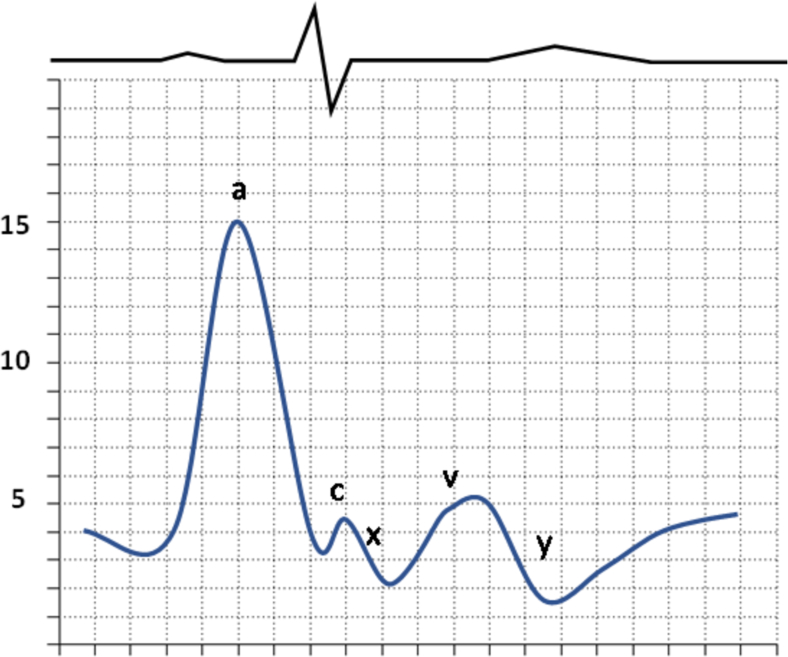
Cannon A Waves (The cannon a wave are seen with with atrioventricular dissociation when the RA contracts against a closed tricuspid valve. These can be seen with ventricular tachycardia or complete heart block.).

**Fig. 3:**
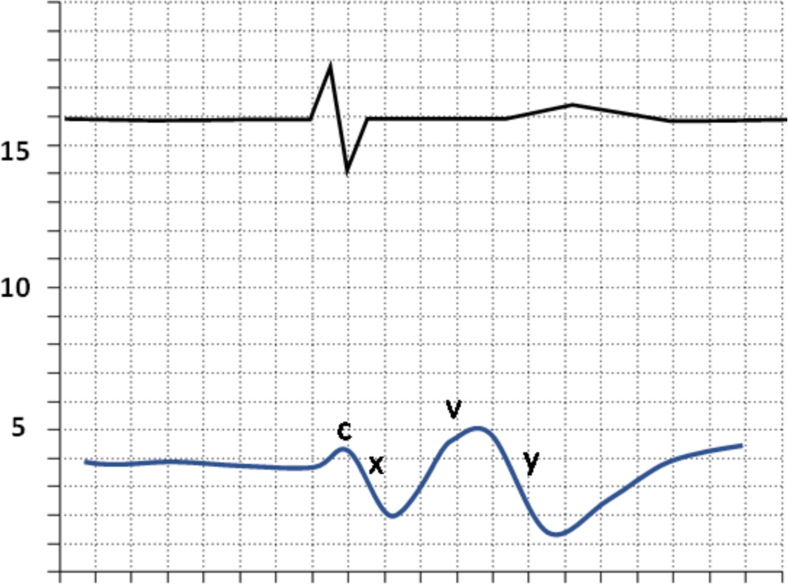
Loss of A Wave (The loss of a wave is seen with a loss of atrial contractions, primarily in the setting of atrial fibrillation.).

**Fig. 4:**
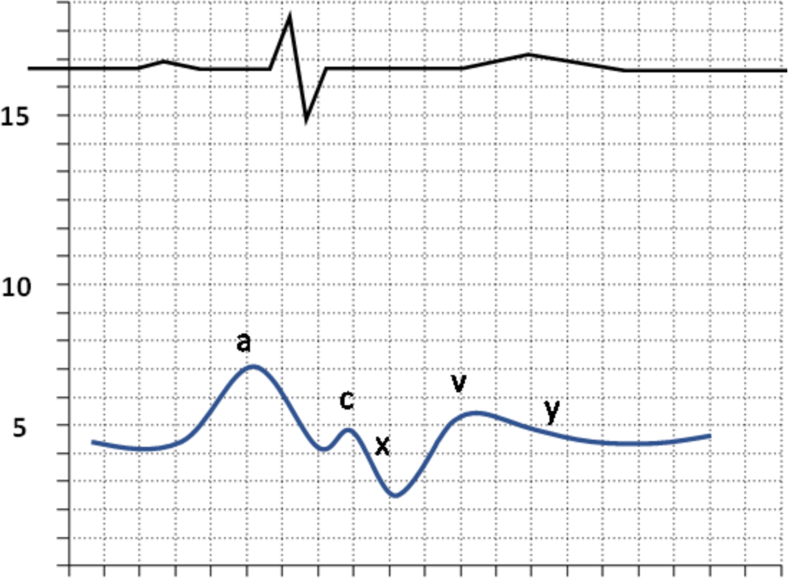
Blunted Y Descent (The blunted y descent occurs in the setting of elevated RV pressures and in the following conditions: tamponade or tricuspid stenosis).

**Fig. 5:**
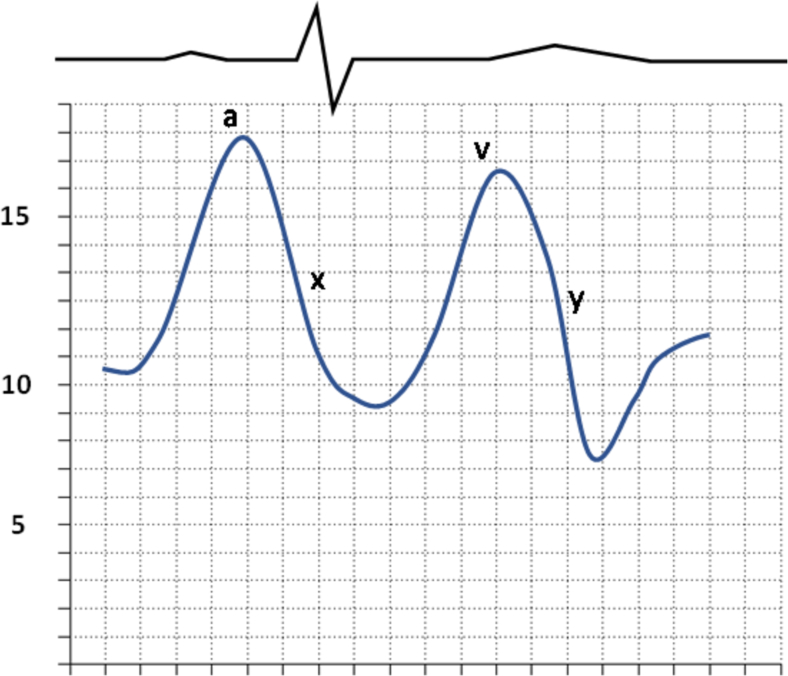
Prominent X and Y Descent (A prominent x descent is found in constrictive pericarditis or cardiac tamponade. The y descent is prominent in found in constrictive pericarditis.).

**Fig. 6:**
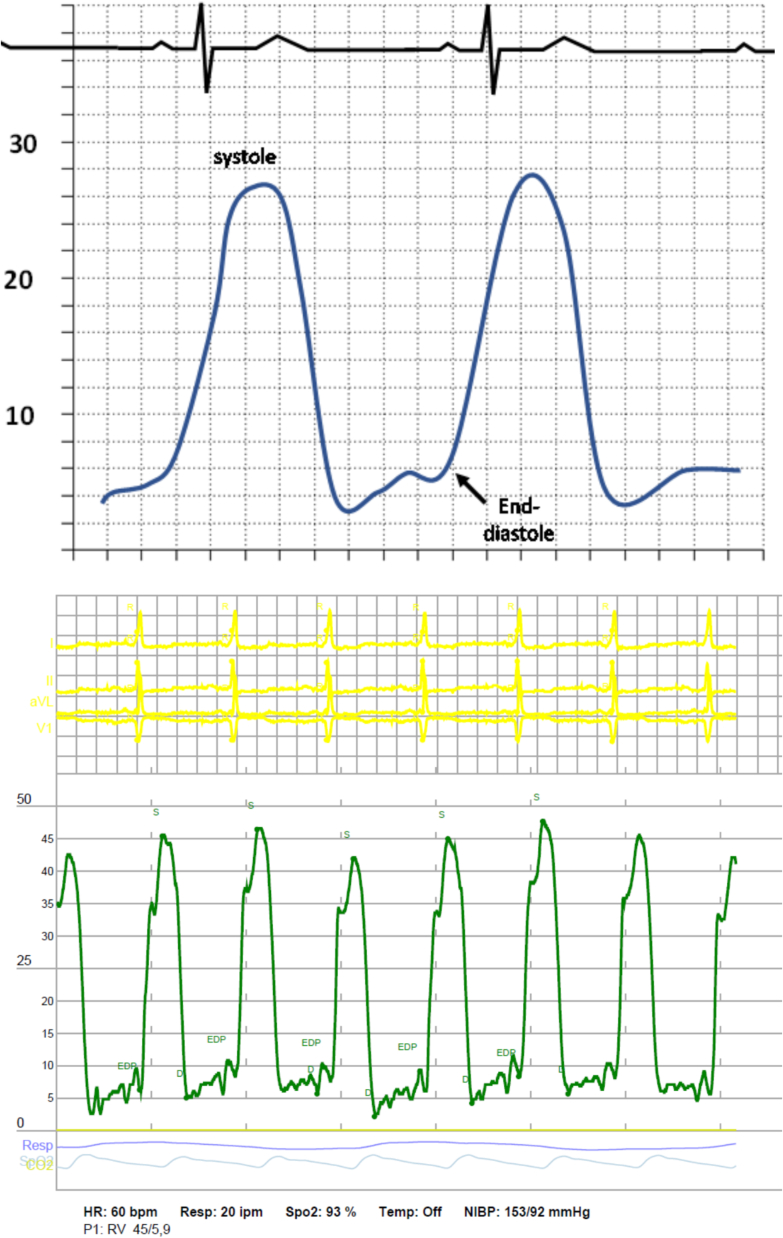
RV Pressure Waveform (The RV pressure waveform is shown above. Normal pressure ranges of the RV during systole are 15–25 mmHg while pressures during diastole range from 1 to 8 mmHg.).

**Fig. 7:**
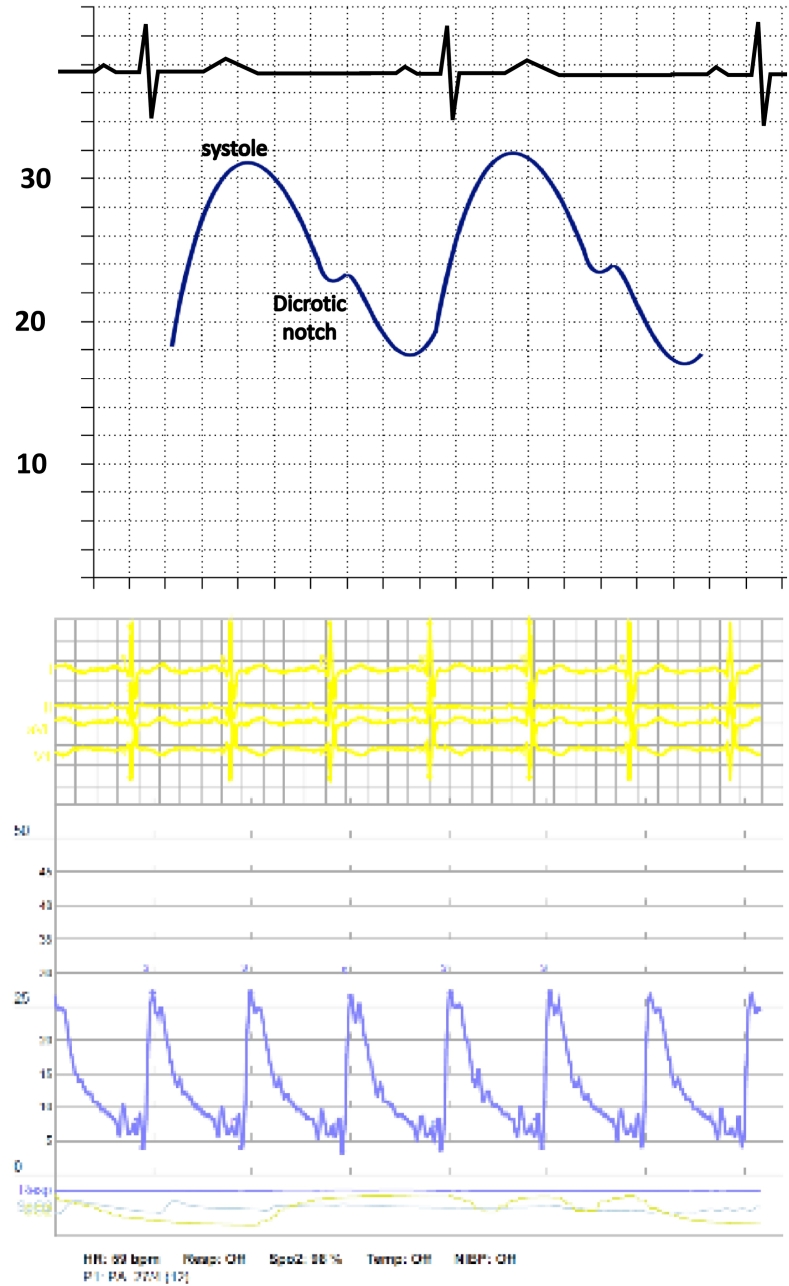
PA Pressure Waveform (The PA diastolic pressure will be representative of the PAWP. The dicrotic notch is when the pulmonic valve closes.).

**Fig. 8:**
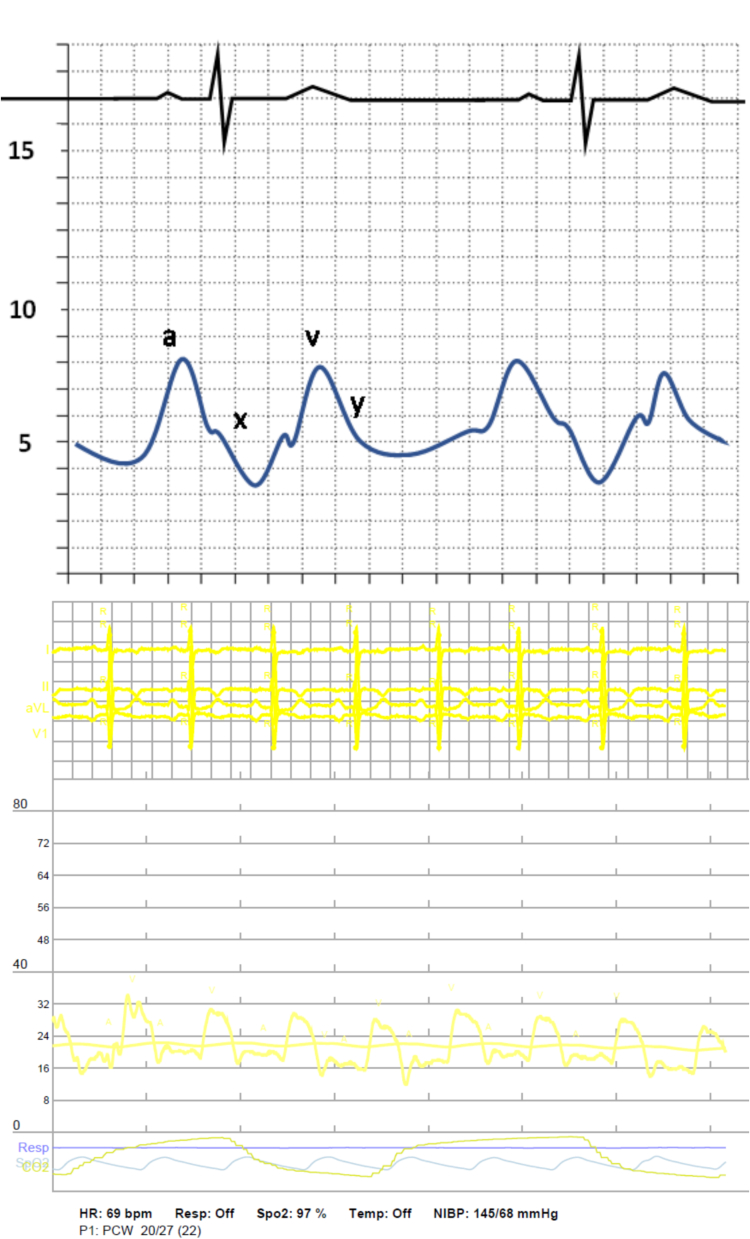
PAWP Waveform (This tracing represents the PAWP. Normal pressure ranges from 6 to 15 mmHg. The a wave represents left atrial contraction, and the v wave is associated with ventricular systole causing a rise in LA pressure. The x descent represents atrial relaxation, and passive filling of the LV is represented by the y descent.).

**Fig. 9:**
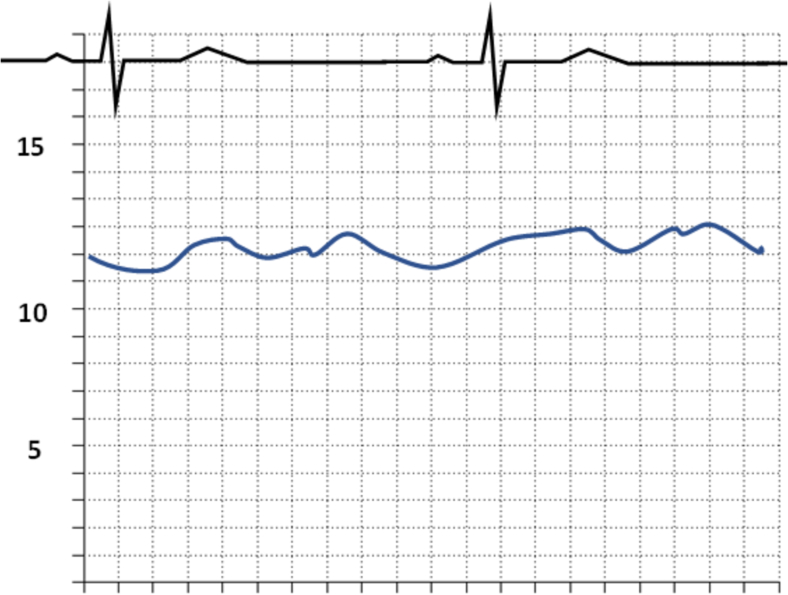
Overwedge PAWP (Overwedging of the PAWP leads to falsely elevated pressures. The pressure waveform may become non-pulsatile as well).

**Fig. 10:**
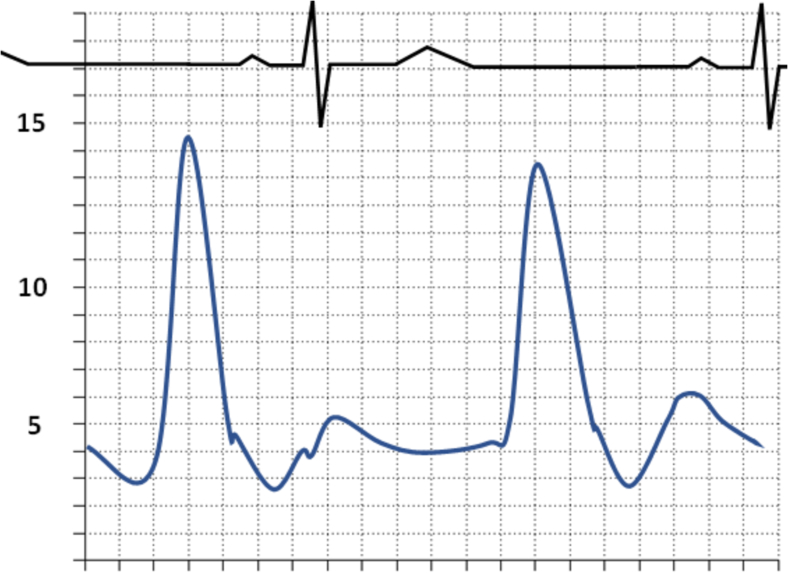
Underwedge PAWP (Under-wedging occurs in the setting of incomplete occlusion of the PA and leads to falsely elevated PAWP resulting in misdiagnosis of PH.).

**Fig. 11:**
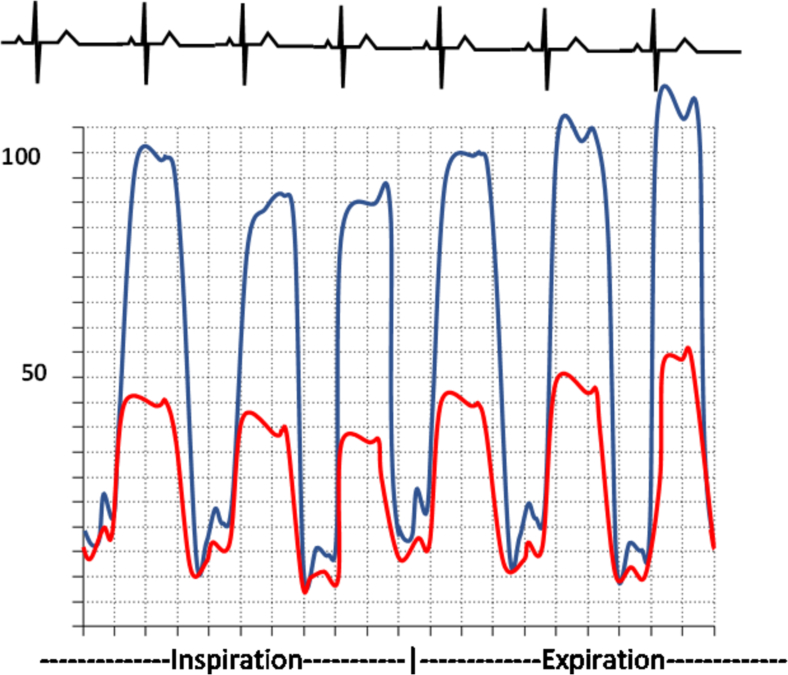
Waveform of Restrictive Cardiomyopathy (Restrictive cardiomyopathy shows concordance of ventricular pressures during respiratory cycle. The rapid y-descent can also be noted.).

**Fig. 12:**
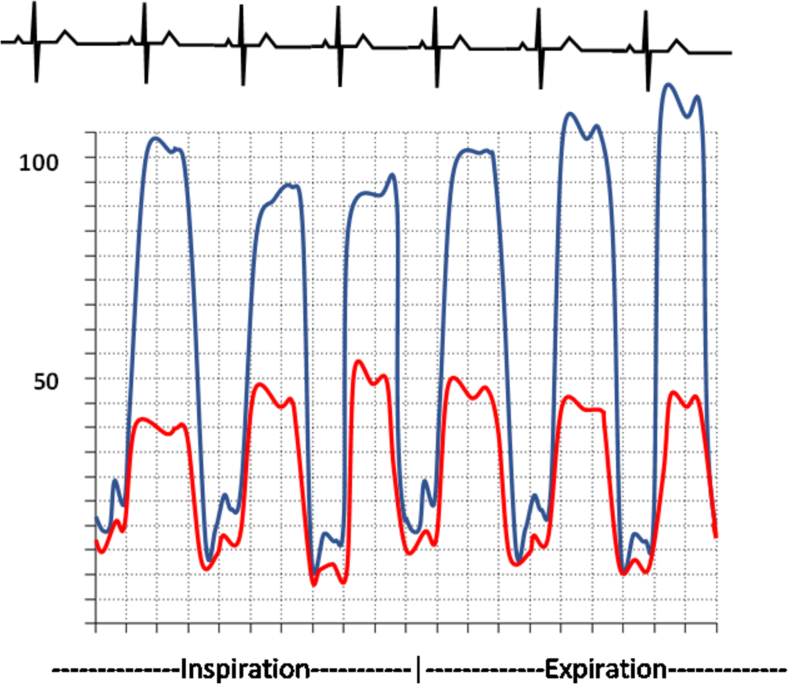

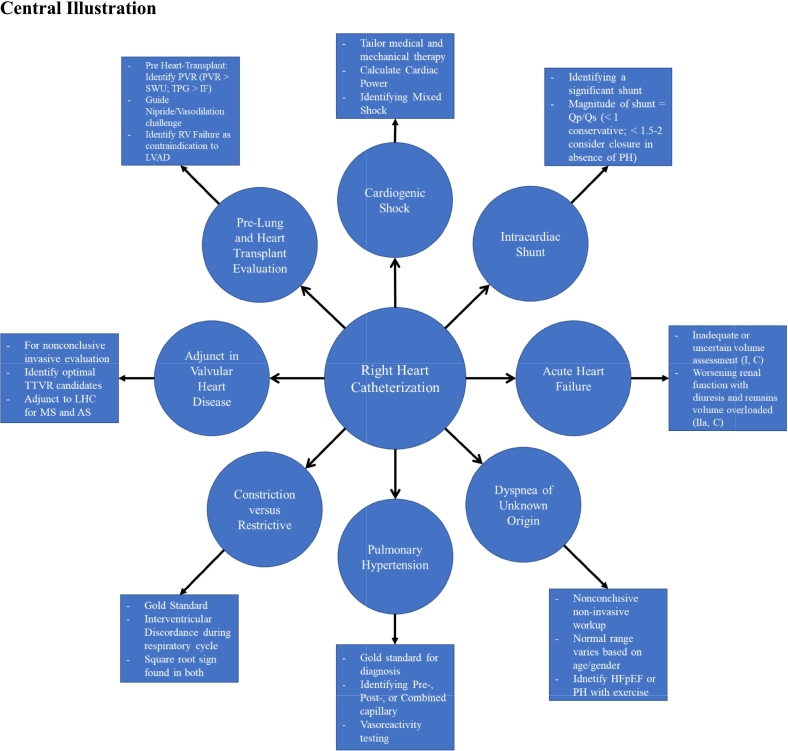
Waveform of Constrictive Disease (Ventricular discordance is found during the respiratory cycle in constrictive physiology. The square root sign can be found as a result of the rapid y-descent.).

Consensus statements have recommended measurement of blood oxygen saturation from the distal PAC tip in the wedge position if PAWP is elevated or if its accuracy is questionable [83]. A arterial oxygen saturation level would indicate true wedge position, while a lower than expected value would indicate incomplete occlusion of the PA. Viray et al. performed a prospective trial to determine the differences in PAWP values and PH classification utilizing oxygen saturation. Oxygen saturation was measured when PAWP measurements were >15 mmHg and occlusive saturation was defined as SaO2 > 90 % or within 5 % of systemic arterial saturation. If these criteria were not met, two additional attempts were made. The authors found that even with confirmation of PAWP by fluoroscopy and appropriate waveform appearance, an occlusive SaO2 was only obtained 50 % of the time. They found that 29 out of 45 (64.4 %) had significantly lower PAWP obtained with position confirmed by SaO2 compared to initial PAWP measurement, and 10 patients were reclassified - 6 changed from post-capillary to pre-capillary PH and 4 changed from post-capillary PH to combined pre- and post-capillary PH [84]. Measurements and calculations can be referenced in Table 6, Table 7, and waveforms obtained from RHC are in Fig. 1, Fig. 2, Fig. 3, Fig. 4, Fig. 5, Fig. 6, Fig. 7, Fig. 8, Fig. 9, Fig. 10, Fig. 11, Fig. 12.

**Table 6:** Direct hemodynamic measurements obtained from RHC.

Hemodynamic measurement	Normal Range	Comments
Central Venous Pressure	2–6 mmHg	
Right atrial pressure	2–6 mmHg	
Right Ventricular Pressure (systolic and diastolic)	Systolic: 15–25 mmHg Diastolic: 1–8 mmHg	
Pulmonary artery pressure (systolic/diastolic/mean)	Systolic: 15–25 mmHg Diastolic: 4–12 mmHg Mean: 9–18 mmHg	
Pulmonary Artery Wedge Pressure	6–15 mmHg	Recorded at end expiration, mean of three to five measurements
Cardiac Output	4–6 L/min	Determined using Thermodilution or Fick
Mixed Venous Oxyhemoglobin Saturation (SvO_2_)	60–75 %

**Table 7:** Hemodynamic Measurements calculated from RHC measurements:

Hemodynamic measurement	Calculation	Normal values
Systemic Vascular Resistance (SVR)	$\frac{\left(\text{mean systemic artery pressure}–\mathrm{CVP}\right)}{\mathrm{CO}}$	700–1600 dynes-*sec*/cm^5^
Pulmonary Vascular Resistance (PVR)	$\frac{\left(\text{mPAP}–\text{PAWP}\right)}{\mathrm{CO}}$	20–120 dynes-sec/cm^5^
Cardiac Index (CI)	$\frac{\mathrm{CO}}{\mathrm{BSA}}$	2.5–4.0 L/min/M^2^
Stroke Volume Index [99]	$\frac{\mathrm{CI}}{\mathrm{HR}}\times 1000$	≥ 35 mL/m^2^
Right Ventricular Stroke Work Index (RVSWI) [99]	$\frac{\left(\text{mPAP}–\mathrm{RAP}\right)\times \mathrm{CI}\times 0.0136}{\mathrm{HR}}$	5–10 g/m^2^/beat
Cardiac Power Output	CPO = (mean arterial pressure − RAP) × cardiac output	1.0 W
Transpulmonary Gradient (TPG) [99]	mPAP – PAWP	< 12 mmHg
Diastolic Pulmonary Gradient (DPG) [99]	Diastolic PAP – PAWP	< 7 mmHg
Pulmonary Artery Pulsatile Index [99]	$\frac{\text{systolic}\;\mathrm{PAP}–\text{diastolic}\;\mathrm{PAP}}{\mathrm{RAP}}$	PAPi <1.85 RV failure after LVAD PAPi <1.0 RV failure in acute MI
Oxygen Delivery	CO × CaO_2_ = CO × [(Hgb × SaO_2_ × 1.34) + (PaO_2_ × 0.0031)]	1000 mL of O_2_/min
Oxygen Uptake	1.3.4 × [CO × Hgb × (SaO_2_ – SvO_2_)]	

CVP = central venous pressure, CO = cardiac output, mPAP = mean pulmonary artery pressure, PAWP = pulmonary artery wedge pressure, BSA = body surface area, CI = cardiac index, HR = heart rate, RAP = right atrial pressure, PAP = pulmonary artery pressure, CaO_2_ = arterial O_2_ content, Hgb = hemoglobin, SaO_2_ = oxygen saturation, PaO_2_ = partial pressure of arterial oxygen, SvO_2_ = mixed venous oxygen saturation

### 8.2 Interpretation of results

PVR (Table 7) is a useful measurement to guide diagnostics and decision making for PAH, subtypes of PH, and heart transplantation. It provides important prognostic information about mortality in PH and heart transplantation patients. Pre-capillary PH is defined as mPAP >20 mmHg, PAWP ≤15 mmHg, and PVR ≥ 2 WU, while post-capillary PH consists of mPAP >20 mmHg, PAWP >15 mmHg, and PVR < 2 WU [85]. Some data have recommended a PVR cut-off of 2.2 WU to differentiate subtypes, and values greater or equal to 2.2 have shown increased mortality [85].

Once diagnosis is established, pulmonary vasoreactivity testing is recommended for idiopathic PAH, heritable PAH, or drug-induced PAH. A positive response is defined as a reduction of mPAP ≥ 10 mmHg to reach an absolute value of mPAP ≤ 40 mmHg with an increased or unchanged CO. Responders are suitable for calcium channel blocker (CCB) therapy and long-term response is defined by New York Heart Association Functional Class I or II and sustained hemodynamic improvement after at least 1 year on CCB therapy alone [85].

Pulmonary effective arterial elastance (Ea) is regarded as a measure of total right ventricular afterload and represents both resistive and pulsatile components. In a study by Tampakakis et al., both Ea and pulmonary artery compliance were better predictors of mortality and right ventricular dysfunction than PVR or TPG in patients with PH due to left heart disease. The authors suggested these parameters could be utilized for therapies targeted towards RV load [86].

### 8.3 Pitfalls in measurement and interpretation

Although RHC provides data and insights that cannot be obtained from clinical exam, it is subject to variability and errors in measurement and interpretations. These errors can lead to misclassification of PH group resulting in inappropriate therapy in patients. The first step to avoid errors in measurement is to ensure the correct set-up of pressure lines and transducers. The pressure transducer should be zeroed at the mid-thoracic level, halfway between the anterior sternum and bed surface while the patient is lying supine. This represents the location of the left atrium, and deviations from can lead to alterations in measurement [21,64]. PAWP should be measured at the mid a-wave of end-expiration with normal breathing (reference Tedford and/or others). Pressures are usually recorded as an average of three to five measurements [16]. The PAWP will accurately reflect LA pressure when the catheter is located in West Zone III (define) where arterial pressure > venous pressure > alveolar pressure in the lung. This position allows for the best indicator of the LA pressure as it creates a continuous column of blood from the catheter directly to the LA. The alveolar pressure will be measured if the catheter ends up in Zone I or II, often signified by absence of a- and v-waves or PA diastolic pressure exceeding PAWP [16].

In patients with chronic obstructive pulmonary disease, increases in intrathoracic pressure during end-expiration can lead to errors in mPAP and PAWP, and measurements should be averaged over the entire respiratory cycle [87]. This may also be the case in obesity, although this should be interpreted with caution as obesity is strongly associated with HFpEF [88]. While these observations were made using an esophageal pressure-transducing catheters as a surrogate of intrathoracic pressures, this technique is unlikey to be routinely employed [83,87,88]. Positive end-expiratory pressure (PEEP) and mechanical ventilation affect RHC hemodynamic values. PEEP leads to increase in pleural and transpulmonary pressures causing reduced RV filling and increased RV afterload. This contributes to an elevated PAWP and overestimation of the LA pressure. Measurement for patients on positive pressure ventilation should be at end-inspiration.

Over-wedging or under-wedging can lead to erroneous PAWP measurements. Over-wedging occurs from excessive inflation of the balloon and makes values falsely low or high. When a catheter balloon is over-wedged, deflating the balloon usually fixes the issue. Over-wedging can lead to increased risk of PA rupture. Under-wedging occurs when the balloon is not completely occluding the PA and provides an artificially elevated measurement. This can be solved by deflating and then inflating the catheter until it wedges completely or sending it into a different PA branch [89].

### 8.4 Cardiac output measurements

Cardiac output (CO) can be measured by either Thermodilution (Td) or Fick methods. Td is obtained by injecting a fluid bolus into the right atrium, then measuring the temperature change of the fluid once it reaches the PA. This change in temperature is plotted against time, and the area under the curve is inversely related to CO [90]. Inaccuracies with Td can occur in patients with severe tricuspid regurgitation, intracardiac shunts, arrythmias, and low CO states [58,91,92]. The estimated oxygen uptake Fick (eFick) method estimates oxygen consumption (V̇O_2_) using body surface area, age, gender, and heart rate, but it can be altered in the setting of HF, PH or abnormal body habitus [93]. In a study comparing measured to estimated V̇O_2_, Narang et al. found that estimated V̇O_2_ is inaccurate leading to a proportional error when deriving CO [94]. Opotowsky et al. studied the correlation between eFick and Td in 15,000 patients undergoing RHC and found that CO estimates differed by >20 % in one-third of patients. Low CO/CI measured by Td was a stronger predictor of mortality compared to eFick. Therefore, Td remains the preferred method for CO assessment as it is a better predictor of all-cause mortality and prognosis in HF and PAH. Furthermore, even in the setting of tricuspid regurgitation, Td measurements were associated with mortality while eFick was not [93].

### 8.5 Pulmonary artery catheterization in the CCU

When the type of shock remains undetermined or there are signs to suggest multi-factorial shock, invasive hemodynamic data can be critical in timely diagnosis and monitoring therapy. Although routine use of PAC after ST segment elevation myocardial infarction (STEMI) is not recommended, it is recommended in patients with CS or hypotension after infarction to determine additional therapy and response. Overall 30-day mortality for CS associated with STEMI remains around 40–50 %, therefore recognition of those at risk is of utmost importance [95]. Multi-factorial shock is particularly challenging to identify without hemodynamic data, and analysis of the patients in the SHOCK (Should we emergently revascularize Occluded Coronaries for cardiogenic shocK) revealed that 18 % of patients with CS showed signs of severe systemic inflammation manifesting as distributive shock. A reduced SVR was found to occur early during shock, prior to the diagnosis of sepsis, which suggested that vasodilation plays an important role in CS [96]. In the SHOCK Trial Registry, cardiac power output (CPO) and cardiac power index (CPI) were independently associated with in-hospital mortality. CPO is calculated as the mean arterial pressure x CO/451, and a CPO ≤0.53 W had a sensitivity and specificity of 66 % for in-hospital mortality [97]. Therefore, the use of RHC can provide additional data if patients do not present or respond to therapies in a classical pattern (Table 8, Table 9, Table 10).

**Table 8:** Calculations of Ea and PAC [86]:

Hemodynamic Measurement	Calculation
Pulmonary effective arterial Elastance (Ea)	End-Systolic PAP Stroke Volume
Pulmonary Arterial Compliance (PAC)	Stroke Volume Pulmonary Artery Pulse Pressure

**Table 9:** Cardiogenic shock data interpretation [14,[100], [101], [102]].

	Right Ventricular Dominant	Left Ventricular Dominant	Biventricular Dominant
Variables			
CVP	> 15 mmHg	< 15 mmHg	> 15 mmHg
PAWP	< 18 mmHg	> 18 mmHg	> 18 mmHg
CVP/PAWP	> 0.8	< 0.8	> 0.8
PAPI	< 1.5	> 1.5	< 1.5
CI	< 2.2 L·min^−1^·m^−2^	< 2.2 L·min^−1^·m^−2^	< 2.2 L·min^−1^·m^−2^
CPO	< 0.6	< 0.6	< 0.6

**Table 10:** SCAI classification of cardiogenic shock (adapted) [10].

	Description	Typical Physical exam/bedside findings	Typical Biochemical markers	Typical Hemodynamics
Stage				
A (at risk)	A patient who is not currently experiencing signs or symptoms of CS, but is at risk for its development	Normal JVP Warm and well perfused	Normal lactate	Normotensive
B (beginning CS)	A patient who has clinical evidence of hemodynamic instability (including relative hypotension or tachycardia) without hypoperfusion	Elevated JVP Warm and well-perfused	Normal lactate	Hypotension (SBP < 90 mmHg, MAP<60 mmHg or > 30 mmHg drop from baseline) Tachycardia (HR > 100 bpm)
C (classic CS)	A patient who manifests with hypoperfusion and who requires one intervention(pharmacological or mechanical)beyond volume resuscitation	Volume overload	Lactate ≥ 2 mmol/L	Cardiac index<2.2 L/min/m2 PAWP>15 mmHg
D (deteriorating/doom)	A patient who is similar to category C but is getting worse. Failure of initial support strategy to restore perfusion as evidenced by worsening hemodynamics or rising lactate	Any of the C and worsening (or not improving) signs/ symptoms of hypoperfusion despite the initial therapy	Any of the stage C and lactate rising and persistently ≥ 2 mmol/L	Any of stage C and requiring escalating doses or increasing numbers of pressors or addition of a mechanical circulatory support device to maintain perfusion
E (extremis)	Actual or impending circulatory collapse	Typically unconscious	Lactate ≥ 8 mmol/L	Profound hypotension despite maximal hemodynamic support

## CRediT authorship contribution statement

**Bhavesh Katbamna:** Writing – review & editing, Supervision. **Lingling Wu:** Writing – review & editing. **Mario Rodriguez:** Writing – review & editing. **Phillip King:** Writing – review & editing, Visualization. **Joel Schilling:** Writing – review & editing, Supervision. **Jamal Mahar:** Writing – review & editing. **Ajith P. Nair:** Writing – review & editing, Supervision. **Hani Jneid:** Writing – review & editing, Supervision. **Elizabeth S. Klings:** Writing – review & editing, Supervision. **Gerald L. Weinhouse:** Writing – review & editing, Supervision. **Sula Mazimba:** Writing – review & editing, Supervision. **Marc A. Simon:** Writing – review & editing, Supervision. **Markus Strauss:** Writing – review & editing, Supervision. **Chayakrit Krittanawong:** Writing – review & editing, Writing – original draft, Visualization, Validation, Supervision, Conceptualization.

## Ethical statement

No ethical statement is neccessary.

## Declaration of competing interest

The authors declare that they have no known competing financial interests or personal relationships that could have appeared to influence the work reported in this paper.
